# *APOE* 5’UTR Methylation Pattern Analysis in Blood and Brain Tissue from Alzheimer’s Disease Affected Patients

**DOI:** 10.14336/AD.2024.0350

**Published:** 2025-06-24

**Authors:** Rosalinda Di Gerlando, Francesca Dragoni, Bartolo Rizzo, Riccardo Rocco Ferrari, Elisabetta Zardini, Matteo Cotta Ramusino, Giulia Perini, Alfredo Costa, Tino Emanuele Poloni, Orietta Pansarasa, Annalisa Davin, Stella Gagliardi

**Affiliations:** ^1^Department of Biology and Biotechnology “L. Spallanzani”, University of Pavia, 27100 Pavia, Italy.; ^2^Molecular Biology and Transcriptomics Unit, IRCCS Mondino Foundation, 27100 Pavia, Italy.; ^3^Department of Brain and Behavioral Sciences, University of Pavia, 27100 Pavia, Italy.; ^4^Laboratory of Neurobiology and Neurogenetics, Golgi-Cenci Foundation, 20081 Abbiategrasso, Italy.; ^5^Neuroimmunology Research Unit, IRCCS Mondino Foundation, 27100 Pavia, Italy.; ^6^Behavioral Neurology Unit, IRCCS Mondino Foundation, 27100 Pavia, Italy.; ^7^Department of Neurology and Neuropathology, Golgi-Cenci Foundation, 20081 Abbiategrasso, Italy.; ^8^Cellular Models and Neuroepigenetics Unit, IRCCS Mondino Foundation, 27100 Pavia, Italy.

**Keywords:** Alzheimer’s disease, *APOE*, methylation, PBMCs, hippocampus

## Abstract

*APOE* ɛ4 allele is the major genetic risk factor for Alzheimer’s Disease (AD). Furthermore, *APOE* methylation pattern has been described to be associated with the disease and to follow a bimodal pattern, with a hypermethylated CpG island and a hypomethylated promoter region. However, little is known about the methylation levels in the *APOE* 5’UTR region. Here, the methylation of two regions (R1 and R2) within *APOE* 5’UTR was investigated in both peripheral blood mononuclear cells (PBMCs) and hippocampus (HIC) samples to identify differentially methylated CpG sites and to associate clinical, genetic features and cerebrospinal fluid (CSF) biomarkers levels. DNA was extracted from PBMCs of 20 AD and 20 healthy controls (HC) and from 6 AD and 3 HC HIC samples. The methylation analysis was carried out by means of pyrosequencing. In AD PBMCs we found that R1 region displayed a higher methylation level, while the opposite trend was observed in R2. The presence of ɛ4 allele highlighted a marked increase in R1 methylation level and a decrease in R2. In AD PBMCs and HIC, age progression resulted to be associated with an increase in the methylation level of R1. Lastly, the methylation of a CpG site in R2 was found to be related to CSF biomarkers. Despite the lack of a statistical significance, the outcome from this exploratory analysis highlighted the presence of a difference in methylation in *APOE* 5’UTR in PBMCs of AD patients which seemed to be associated also with *APOE* genotype, age and CSF biomarkers level.

## INTRODUCTION

Alzheimer’s disease (AD) is the worldwide leading cause of progressive cognitive impairment and dementia in individuals older than 65 years of age [1]. AD is commonly classified as either late onset (LOAD) or early onset (EOAD) which account for 90% - 95% and 5% - 10% of all cases respectively [2,3]. Different factors are associated with an increased risk to develop AD [4]; among them aging is the first one [5], followed by the genetic factors [6] and the presence of comorbidities such as cerebrovascular diseases [7], diabetes [8] and obesity [9].

Among genetic factors, *APOE* is the strongest one associated with LOAD [10]. *APOE* gene is located on chromosome 19; it comprises 4 exons and it encodes for apolipoprotein E (ApoE) [11]. In the Central Nervous System (CNS) ApoE is mainly produced by astrocytes and microglia and it covers different functions, among which the regulation of lipid transport, synaptic function, neuroinflammation, and the metabolism and clearance of amyloid-β (Aβ) monomers [12]. This last process has been described to be affected by ApoE isoform [13]. Importantly, on *APOE* exon 4 there are two single-nucleotide polymorphisms (SNPs, rs429358 and rs7412) the combination of which gives rise to three alleles (ε2, ε3 and ε4), that in turn encode for three different protein isoforms (ApoE2, ApoE3 and ApoE4) [14]. Depending on the genotype, the risk to develop AD increases up to 4-fold if one copy of ε4 allele is present and up to 15-fold if two copies of ε4 are present [15]. As a direct consequence, it has been observed that Aβ clearance is affected in an isoform-dependent manner, with ApoE4 having the greatest influence on clearance reduction followed by ApoE3 and then ApoE2 [16]. Interestingly, *APOE* CpG island is also located on exon 4 and the combination of the two SNPs was observed to also affect the methylation pattern of this gene region [17].

Over the past years, a growing number of studies have highlighted an association between methylation variation and AD pathology [18]. On this basis, different epigenome-wide associations studies (EWAS) have identified several genes and regions that appear to be differentially methylated in people affected by AD [19,20]. Namely, *TREM2* gene was described to have a disease-associated hypermethylated region in AD patients’ superior temporal gyrus [21]. Some years later, the same group also found a significant hypermethylation and hypohydroxymethylation in *ANK1* gene in entorhinal cortex of AD patients [22]. Another interesting study conducted on different AD brain regions showed a hypermethylation and increased expression of *APP* and *MAPT*, the two genes at the basis of AD molecular pathology [23].

More recently, the idea of using newly-identified differentially methylated regions as new peripheral blood-based AD biomarkers has come forward [24]. In this context, it was observed that the loci displaying an aberrant methylation were mostly enriched in brain and neurodegeneration-related genes [24]. Interestingly, a follow-up study performed on a cohort of cognitively healthy individuals aged over 70 years found that in particular three genes, *APP, APOE* and *TOMM40*, displayed different blood DNA methylation levels between individuals subsequently diagnosed with dementia and individuals that remained cognitively healthy [25]. Given *APOE* role in the pathology, a number of studies have tried to unravel the core mechanism that marks this gene as a major AD genetic risk factor, and many focused on its methylation pattern, mostly of its CpG island [17,26]. Although the lack of a unanimous consent in the literature, *APOE* gene methylation was described to follow a bimodal pattern in both prefrontal cortex and lymphocytes, with a hypomethylated promoter region and a hypermethylated CpG island located on exon 4 [27]. The hypomethylation at *APOE* promoter region as well as its association with the disease in hippocampus and lymphocytes of AD patients was also observed in another study [28].

Starting from this and considering the limited and often conflicting data on the methylation of *APOE* regulatory region, this study aimed at assessing a putative differential methylation in the region spanning *APOE* 5’UTR and identifying single CpG sites to possibly associate with the disease. Working on both Peripheral Blood Mononuclear Cells (PBMCs) and post-mortem hippocampus (HIC) brain tissue, the relationship between this region methylation and clinical features [28] was also studied.

## MATERIALS AND METHODS

### Patients’ enrollment

Blood samples from 20 AD patients were collected in EDTA tubes at the IRCCS Mondino Foundation, Pavia (Italy). Patients were selected after being examined and diagnosed according to the National Institute on Aging-Alzheimer’s Association (NIA-AA) [29] criteria. Subjects with a typical form of disease onset, minor comorbidities and similar symptomatic spectrum were selected. All subject’s global cognitive status was assessed by the Mini-Mental State Examination (MMSE). Blood samples from 20 HC volunteers were obtained from the Transfusional Service and Centre of Transplantation Immunology, IRCCS San Matteo Foundation, Pavia (Italy). Individuals were selected depending on sex, age, healthy neurological condition and absence of pharmacological therapy. Post-mortem HIC brain tissue samples of 6 AD and 3 HC were provided by Golgi Cenci Foundation, Abbiategrasso (Italy). Donors were all subjected to the same clinical and neuropathological protocol [30]. Both for AD and for HC, brain tissue collection was performed within 24 hours from death and both hemispheres were cut in slices and frozen [30]. Subsequently, HIC sections of about 15 mg were obtained and used for DNA methylation analysis. All subjects involved in this study signed an informed consent form. Details on the subjects included in the study are reported in Table 1, Supplementary Table 1 and 2.

**Table 1:** Characteristics of recruited subjects for this study.

	Blood samples		HIC brain tissue	
*Group*	AD	HC	AD	HC
*Recruited subjects*	20	20	6	3
*Age (mean ± SD)*	72,45 ± 8,8	60,1 ± 6,21	82,33 ± 4, 03	77 ± 5,29
*Sex*				
*Females n (%)*	70%	30%	66,66%	33,33%
*Males n (%)*	30%	70%	33,33%	66,66%

AD = Alzheimer’s Disease. HC = healthy control; HIC = hippocampus SD = standard deviation.

### DNA extraction from whole blood and HIC brain tissue and APOE genotype

Genomic DNA was extracted from 200 µL of whole blood using Maxwell® CSC 48 instrument (Promega, USA) together with Maxwell^®^ CSC Blood DNA Kit (Promega, USA). Maxwell^®^ CSC Genomic DNA Kit (Promega, USA) was used to extract DNA from 15 mg of frozen HIC brain tissue. DNA quantification was obtained with NanoDrop™ One/OneC Microvolume UV-Vis Spectrophotometer (Thermo Fisher Scientific, USA). *APOE* genotype was assessed using TaqMan SNP Genotyping Master Mix (Applied Biosystems, USA) with the CFX384 Touch Real Time PCR Detection System (Biorad, USA). The two polymorphisms have been analyzed using TaqMan™ SNP Genotyping Assay, human (Applied Biosystems, USA): probe C 3084793_20 for rs429358; probe C____904973_10 for rs7412. Fluorescence was read at cycle 32 for the rs429358 assay and at cycle 42 for the rs7412 assay and *APOE* genotype was obtained.

### Isolation of Human Peripheral Blood Mononuclear Cells (PBMCs) and DNA extraction

PBMCs isolation was performed using Histopaque^®^-1077 (Sigma-Aldrich, USA) starting from peripheral venous blood of AD patients (n = 20) and HC subjects (n = 20) and following manufacturer's instructions. DNA was then extracted using the DNeasy Blood and Tissue Kit (Qiagen, Germany) starting from 5x10^6^ PBMCs pellet according to the manufacturer’s guidelines. NanoDrop™ One/OneC Microvolume UV-Vis Spectrophotometer (Thermo Fisher Scientific, USA) was used to determine DNA concentration.

**Table 2:** PCR and Pyrosequencing primer sequences.

	PCR primer sequence	Pyrosequencing primer sequence
**R1**		
*Forward*	TAGGGGATTGGATTTGGGAAGG	GGGTTGGGTAGTAGA
*Reverse*	Bio-ACCCCTAACTCCCCAATT	
**R2**		
*Forward*	AGTAGTTGGATTGGGATGTAAGTT	GGGGAGTTAGGGGTA
*Reverse*	Bio-CCCTTCACATTCTAAACTCCA	

Bio = biotin-labelled primer; R1 = region 1; R2 = region 2.

### APOE 5’UTR methylation analysis

The methylation pattern of *APOE* region encompassing the 5’UTR and the first intron (chr19:44,906,009-44,906,264, GRCh38/hg38) was investigated by means of pyrosequencing technique. Initially, the genomic DNA was treated with sodium bisulfite to allow the conversion of unmethylated cytosines into uracil using EpiTect Bisulfite Kit (Qiagen, Germany) and following manufacturer’s instructions. Then, bisulfited DNA underwent a clean-up in preparation for PCR. To allow the correct formation of amplicons, given its length, the previously described region was divided in two smaller overlapping regions, namely region 1 (R1; chr19:44,905,989-44,906,176, GRCh38/hg38) and region 2 (R2; chr19:44,906,058-44,906,285, GRCh38/hg38). R1 contains 5 CpG sites (1^CpG1^, 1^CpG2^, 1^CpG3^, 1^CpG4^, 1^CpG5^) while R2 contains 6 CpG sites (2^CpG1^, 2^CpG2^, 2^CpG3^, 2^CpG4^, 2^CpG5^, 2^CpG6^). PCR was performed using PyroMark PCR kit (Qiagen, Germany) following manufacturer’s guidelines and two amplicons of 187 bp and 228 bp were obtained, respectively for R1 and R2. Primers were designed using PyroMark Assay Design 2.0 software (Qiagen, Germany) and are listed in Table 2. The quality of the amplicons was checked by gel electrophoresis. Finally, PCR products were sequenced using sequencing primers (Table 2), PyroMark Q48 Advanced CpG Reagents (Qiagen, Germany) and PyroMark Q48 instrument (Qiagen, Germany). The methylation level is represented by the percentage of 5-methylcytosine. For the analysis both the methylation percentage of each site and the average of the methylation percentage of the first three sites were used. The rationale behind the decision to consider only the first three sites stemmed from the fact that during pyrosequencing the CpG sites located at the beginning of the sequence are usually those with the highest quality score. This is due to technical reasons intrinsic to the method.

### RNA extraction and APOE expression analysis

Total RNA was isolated starting from 5x10^6^ PBMCs pellet using Trizol reagent (Life Science Technologies, Italy) according to manufacturer’s instructions. The same procedure was used to extract RNA from 15 mg of cryopreserved HIC samples after a homogenization step performed with the CK14 Precellys lysing Kit (Bertin Technologies, France). NanoDrop™ One/OneC Microvolume UV-Vis Spectrophotometer (Thermo Fisher Scientific, USA) was used to measure RNA concentration and to check its quality; RNAs with A260/A280 ratio ~1.8 and with A260/A230 ratio ranging between 1.8 and 2.2 were considered of good quality. Reverse transcription was achieved using iScript™ cDNA Synthesis Kit (BioRad, USA) and then iQ™ SYBR® Green Supermix (BioRad, USA) was used to perform real time PCR (qPCR). Primer3Plus (version 3.3.0) [31] was used to design qPCR primers (Table 3). *APOE* cycle threshold (Ct) values were normalized against *GAPDH* obtained one. Fold-expression differences between AD and HC groups were determined using the 2^-∆∆Ct^ method.

**Table 3:** qPCR primer sequences.

	qPCR primer sequence
**APOE**	
*Forward*	CTGGCACTGGGTCGCTTTT
*Reverse*	GGGGTCAGTTGTTCCTCCAG
**GAPDH**	
*Forward*	ATGGAAATCCCATCACCATCTT
*Reverse*	CGCCCCACTTGATTTTGG

### Cerebrospinal fluid (CSF) collection and total TAU, pTAU181, Aβ40 and Aβ42 analysis

Cerebrospinal fluid (CSF) samples were collected by lumbar puncture from only a subgroup of the Mondino Foundation AD cohort (n=17) and stored in sterile polypropylene tubes (SARSTEDT AG & Co, Germany). For the remaining 3 AD patients, CSF was not collected. CSF total TAU (tTAU), TAU protein phosphorylated at residue 181 (pTAU181), Aβ40 and Aβ42 were measured using the high-throughput LUMIPULSE^®^ G600II instrument (Fujirebio, Japan) together with Lumipulse^®^ G Total Tau, Lumipulse^®^ G pTau 181, Lumipulse^®^ G β-Amyloid 1-40, Lumipulse^®^ G β-Amyloid 1-42 relative assays (Fujirebio, Japan). CSF Aβ42/Aβ40 ratio was also considered.

### Statistical analysis

GraphPad Prism version 9 (USA) was used to perform statistical analysis and to obtain figures. For both regions the methylation levels were compared between AD and HC groups by means of One-way ANOVA and Student’s t test by considering the methylation percentage of each site and the average of the methylation percentage of the first three sites respectively. The One-way ANOVA with the non-parametric Kruskal-Wallis test was used to compare the mean methylation values of each CpG site within the two regions and between the two groups and check for a significant difference. The Student’s t test with the non-parametric Mann-Whitney test was used to assess whether there was a statistical difference between the mean methylation values of the first three sites of the two regions between AD and HC groups. The One-way ANOVA Kruskal-Wallis test was also performed to assess the effect of the ε4 allele on the methylation percentage of each CpG site within the two regions, while Student’s t Mann-Whitney test was employed to determine effect of the ε4 allele on the average of the methylation percentage of the first three sites within the two regions between AD and HC groups. Student’s t Mann-Whitney test was also used to determine the difference of *APOE* expression between AD and HC groups. A One-way ANOVA Kruskal-Wallis analysis was performed to determine the effect of sex on the average of the methylation percentage of the first three sites between the two groups. Data were presented as mean ± SEM. Finally, Pearson correlation coefficients were used to assess the relationship between the two regions methylation levels, age at sampling and MMSE in the two different groups. The same test was also used to address a possible correlation between the methylation levels of 2^CpG2^ site and the CSF concentration of tTAU, pTAU181, Aβ42 and Aβ42/Aβ40 ratio.

## RESULTS

In this study the methylation pattern of *APOE* 5’UTR region was investigated in PBMCs of 40 subjects, including 20 AD and 20 HC and in HIC brain tissue of 6 AD and 3 HC individuals (Table 1). Sex, age, *APOE* genotype and MMSE score (when available) were set into relationship with the methylation levels measured by bisulfite pyrosequencing, while qPCR was used to assess *APOE* expression levels.

**Figure 1.**
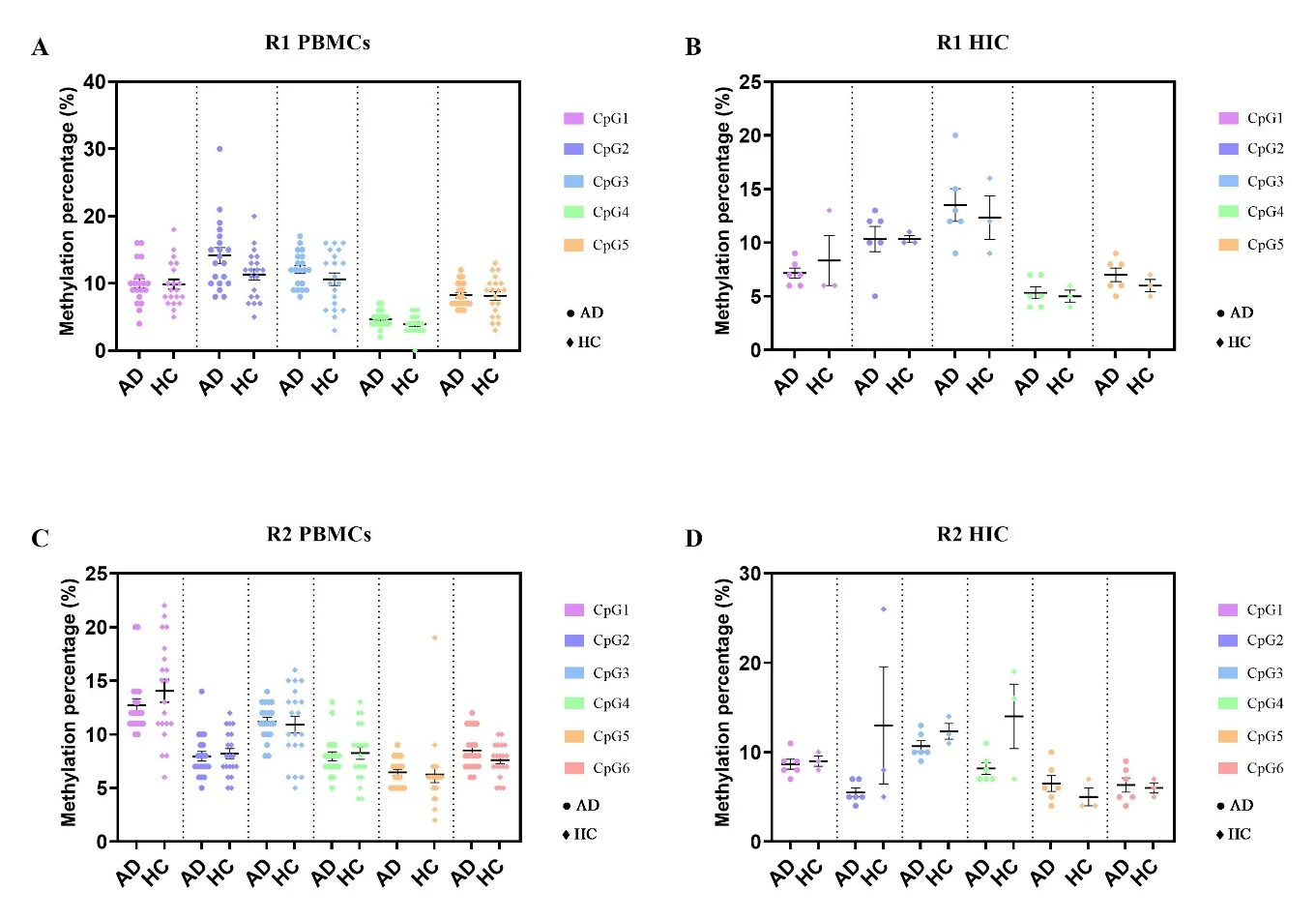
**Methylation analysis of single CpG sites in the two regions comprised in *APOE* 5’UTR of both PBMCs and HIC brain tissue**. (**A**) R1 methylation level in AD (n=20) and HC (n=20) PBMCs. (**B**) R1 methylation level in AD (n=6) and HC (n=3) HIC brain tissue. (**C**) R2 methylation level in AD (n=20) and HC (n=20) PBMCs. (**D**) R2 methylation level in AD (n=6) and HC (n=3) HIC brain tissue. X axis: condition; Y axis: single CpG site methylation percentage. Single CpG sites are differentiated by color. AD and HC groups are differentiated by symbol (● = AD; ♦ = HC). Statistical analysis was performed using the one-way ANOVA Kruskal-Wallis test comparing AD with HC methylation percentage site by site and data are expressed as mean ± SEM. Data are not significant (Supplementary Table 3).

### Analysis of the methylation level of APOE 5’UTR region in AD patients and HC

The methylation level of *APOE* 5’UTR is shown in Figure 1. As previously mentioned, *APOE* 5’UTR has been divided into two smaller regions for technical limitations. For each region, the methylation of single CpG sites were considered and described in both PBMCs and HIC brain tissue. In particular, in PBMCs R1, 1^CpG2^ and 1^CpG3^ showed a higher methylation level in AD with respect to HC subjects (Fig. 1A). A similar trend was observed in the methylation analysis performed on HIC samples, specifically for 1^CpG3^, while for 1^CpG2^ the average methylation percentage was lowered by the score obtained by one AD sample (Fig. 1B). As for R2, in PBMCs, only 2^CpG5^ and 2^CpG6^ displayed a slightly higher methylation percentage in AD, while 2^CpG2^ and 2^CpG4^ showed an opposite trend, with a higher methylation level in HC samples (Fig. 1C). In brain tissue, 2^CpG5^ and 2^CpG6^ methylation resulted higher in AD rather than HC, whereas 2^CpG2^ and 2^CpG4^ maintained the previously described opposite trend between the two conditions, confirming what was observed in PBMCs (Fig. 1D). All data described resulted not statistically significant (details on the statistical analysis with relative p-values are listed in Supplementary Table 3).

### Analysis of the average methylation level of APOE 5’UTR in AD patients and HC

**Figure 2.**
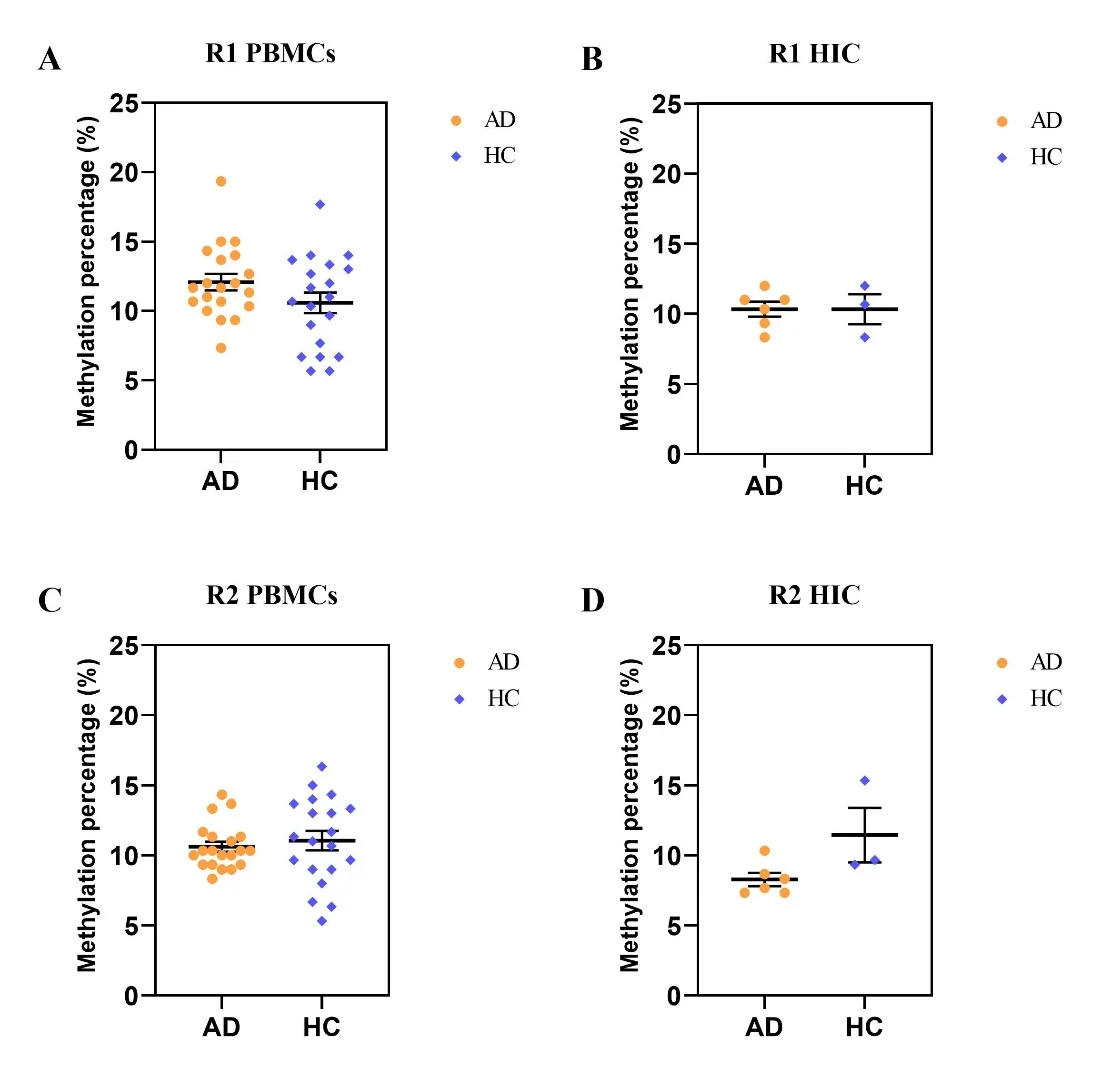
**Average methylation level of the two regions comprised in *APOE* 5’UTR of both PBMCs and HIC brain tissue**. (**A**) R1 average methylation level in AD (n=20) and HC (n=20) PBMCs. (**B**) R1 average methylation level in AD (n=6) and HC (n=3) HIC brain tissue. (**C**) R2 average methylation level in AD (n=20) and HC (n=20) PBMCs. (**D**) R2 average methylation level in AD (n=6) and HC (n=3) HIC brain tissue. X axis: condition; Y axis: average methylation percentage of the first three CpG sites. AD and HC groups are differentiated by color and symbol (● = AD; ♦ = HC). Statistical analysis was performed using the Student’s t test comparing AD with HC methylation percentage and data are expressed as mean ± SEM; Data are not significant (Supplementary Table 4).

To get an overall view on the methylation state of the two regions in the two different tissues, the average methylation percentage of the first three CpG sites was considered and compared between AD and HC (Fig. 2). In PBMCs, R1 average methylation score resulted to be higher in AD than HC subjects, even if in a not significant way (Fig. 2A). The brain tissue analysis highlighted the absence of a difference between the two conditions (Fig. 2B). As for R2, the methylation level appeared to be higher in HC rather than in AD patients in both PBMCs and HIC brain tissue, despite the lack of a statistical significance (Fig. 2C, D) (Supplementary Table 4).

### APOE ε4^-/+^ genotype effect on methylation level of APOE 5’UTR in AD patients and HC

**Figure 3.**
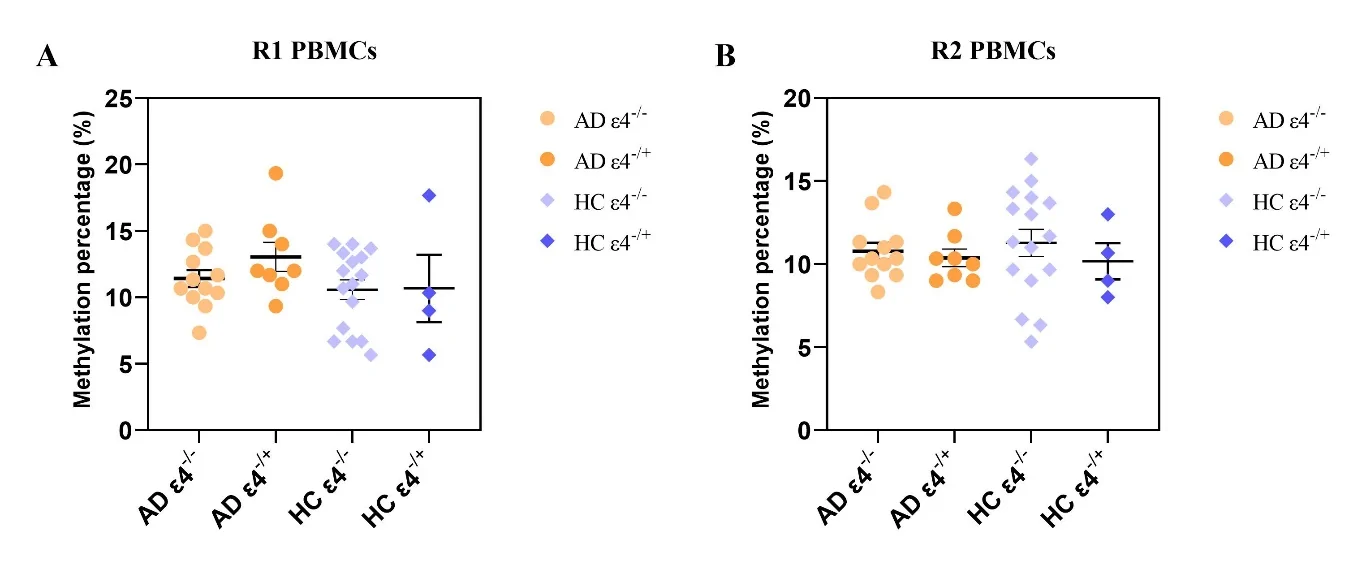
***APOE* ε4 allele effect on average methylation level of the two regions in PBMCs**. (**A**) R1 average methylation level in AD ε4^-/-^ (n=12), AD ε4^-/+^ (n=8), HC ε4^-/-^ (n=16) and HC ε4^-/+^ (n=4) PBMCs. (**B**) R2 average methylation level in AD ε4^-/-^ (n=12), AD ε4^-/+^ (n=8), HC ε4^-/-^ (n=16) and HC ε4^-/+^ (n=4) PBMCs. X axis: condition and *APOE* genotype; Y axis: average methylation percentage of the first three CpG sites. Different genotypes are differentiated by color. AD and HC groups are differentiated by color and symbol (● = AD; ♦ = HC). Statistical analysis was performed using the one-way ANOVA Kruskal-Wallis test comparing all variables and data are expressed as mean ± SEM. Data are not significant (Supplementary Table 5).

*APOE* genotype was assessed for all samples included in this study and its effect on *APOE* 5’UTR methylation was investigated. Given the low number of brain tissue samples, this analysis was conducted only on PBMCs data. Initially, the effect of ε4 allele on the average methylation percentage of the first three CpG sites of the two regions was analyzed in both the AD and the HC groups (Fig. 3). In R1 the presence of ε4 allele determined a not significant increase in the average methylation, markedly in AD but also in HC groups (Fig. 3A). As for the second region, ε4 allele seemed to lower the methylation percentage under both pathological and normal conditions (Fig. 3B). These results appeared to corroborate the influence of the genotype on this *APOE* 5’UTR methylation (Supplementary Table 5).

In a second analysis, AD and HC subjects carrying at least one copy of the ε4 allele were considered; hence, the only considered genotype for both AD and HC was ε4^-/+^ (Supplementary Fig. 1). In R1, the methylation level of all CpG sites of AD patients resulted higher than the HC group (Supplementary Fig. 1A) even if not in a significant way. The same trend was observed also in R2, with the only exception of 2^CpG2^ that showed an opposite trend and 2^CpG4^ that resulted in no difference between the disease and control condition (Supplementary Fig. 1B) (Supplementary Table 6).

### Age effect on methylation level of APOE 5’UTR in AD patients and HC

**Figure 4.**
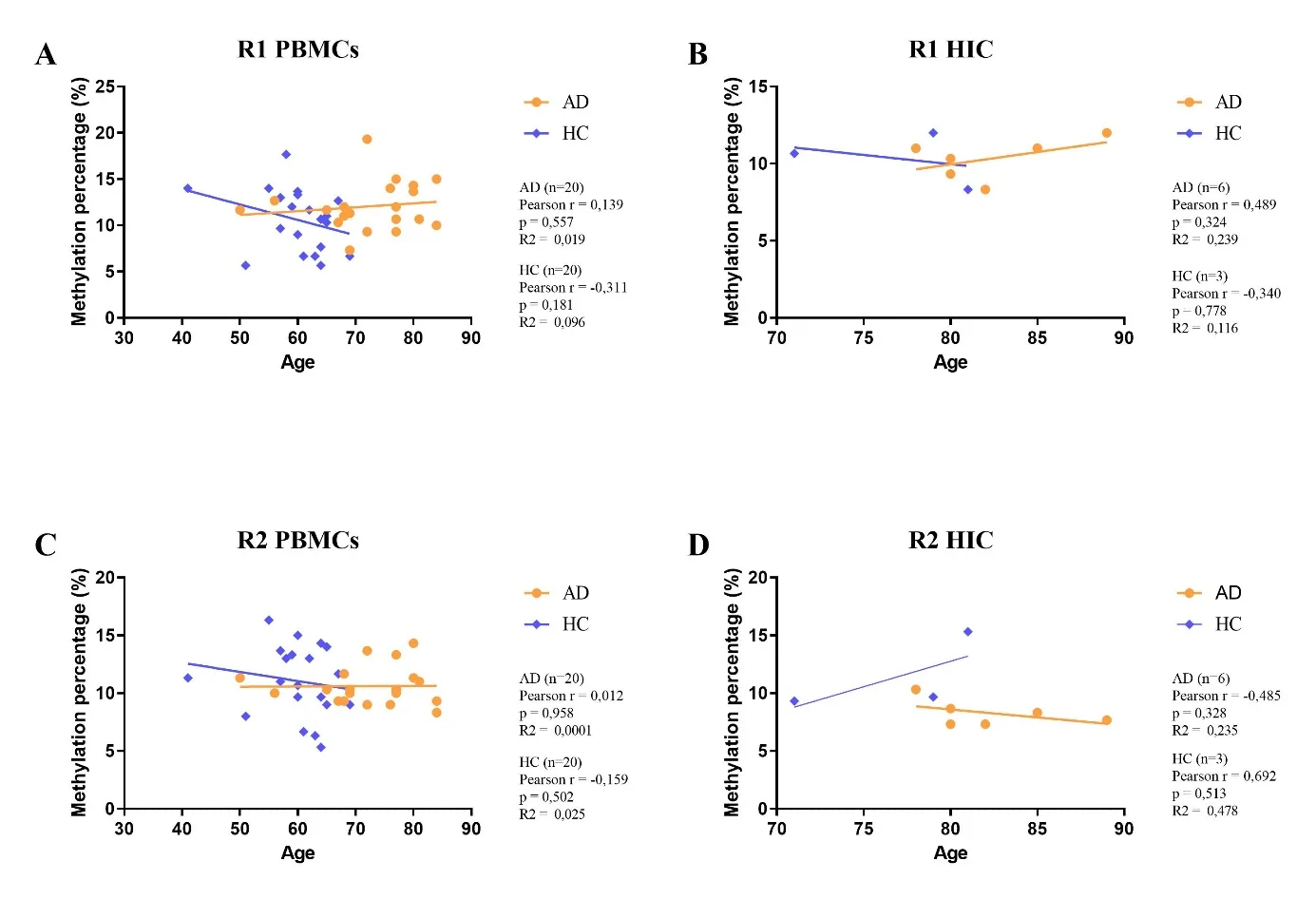
**Correlation between age at sample collection and *APOE* 5’UTR methylation levels**. (**A**) R1 average methylation level in AD group (n=20) and in HC group (n=20) in PBMCs. (**B**) R1 average methylation level in AD group (n=6) and in HC group (n=3) in HIC brain tissue. (**C**) R2 average methylation level in AD group (n=20) and in HC group (n=20) in PBMCs. (**D**) R2 average methylation level in AD group (n=6) and in HC group (n=3) in HIC brain tissue. X axis: age; Y axis: average methylation percentage of the first three CpG sites. AD and HC groups are differentiated by color and symbol (● = AD; ♦ = HC). The relationship between age at the time of sample collection and average methylation level was assessed by Pearson’s correlation coefficient and R square (R^2^).

Considering aging as the main risk factor in AD pathology [5], the correlation between age and *APOE* 5’UTR methylation level was investigated. In this analysis, the average methylation percentage of the first three CpG sites was considered, set into relation with age and compared between AD and HC in both tissues (Fig. 4).

The results obtained in PBMCs showed that in the context of AD pathology, methylation levels tend to increase with increasing age, more in R1 (Pearson r=0.139; p=0.557; R^2^=0.019) than in R2 (Pearson r=0.012; p=0.958; R^2^=0.0001) (Fig. 4A, C). On the contrary, under normal aging condition, methylation was found to decrease with increasing age both in R1 (Pearson r=-0.311; p=0.181; R^2^=0.096) and in R2 (Pearson r=-0.159; p=0.502; R^2^=0.025) (Fig. 4A, C). The analysis performed on brain tissue confirmed what was observed in PBMCs for R1 (AD: Pearson r=0.489; p=0.324; R^2^=0.239; HC: Pearson r=-0.340; p=0.778; R^2^=0.116) (Fig. 4B). R2 showed opposite results, with a decrease in methylation associated with old age in AD and an increase in methylation in HC group (AD: Pearson r=-0.485; p=0.328; R^2^=0.235; HC: Pearson r=0.692; p=0.513; R^2^=0.478) (Fig. 4D).

**Figure 5.**
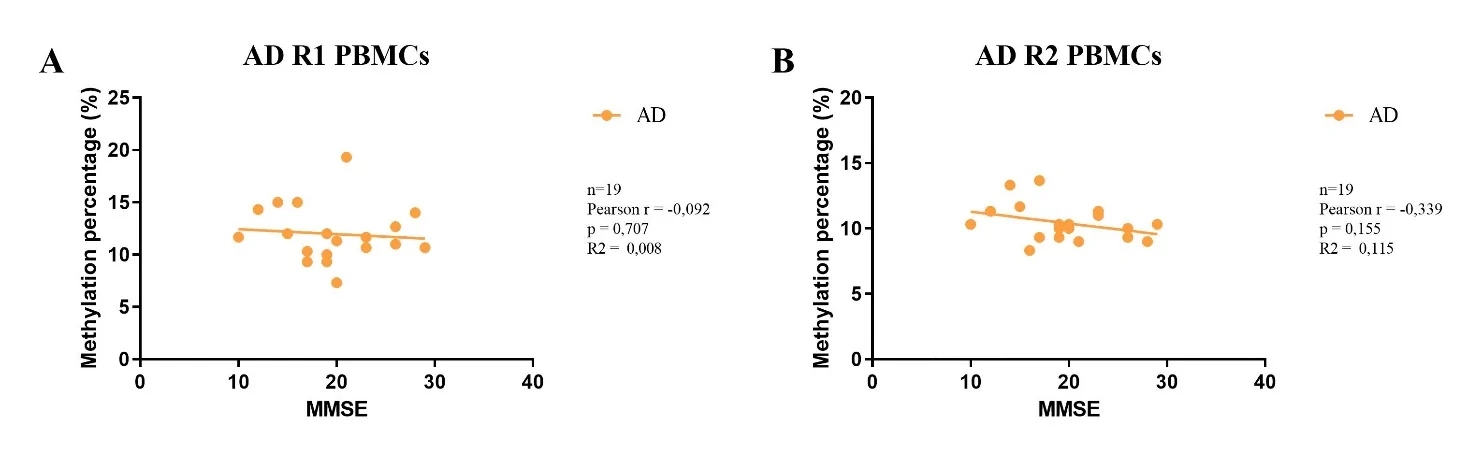
**Correlation between MMSE score and *APOE* 5’UTR methylation levels**. (**A**) R1 average methylation level in AD group (n=19) in PBMCs. (**B**) R2 average methylation level in AD group (n=19) in PBMCs. X axis: MMSE score; Y axis: average methylation percentage of the first three CpG sites. The relationship between MMSE score and average methylation level was assessed by Pearson’s correlation coefficient and R square (R^2^).

### MMSE score effect on methylation level of APOE 5’UTR in AD patients and HC

MMSE score was set into relation with *APOE* 5’UTR methylation level. This analysis was only performed on PBMCs samples since the MMSE test could not be administered to the majority of patients that donated brain tissue samples. The average methylation percentage of the first three CpG sites was correlated to the MMSE score of AD patients for both regions (Fig. 5). With this analysis it was observed that in both regions, the average methylation level decreased with increasing MMSE score, hence with a higher degree of cognitive function (R1: Pearson r=-0.092; p=0.707; R^2^=0.008; R2: Pearson r=-0.339; p=0.155; R^2^=0.115) (Fig. 5A, B).

### Sex effect on methylation level of APOE 5’UTR and APOE expression level in AD patients and HC

The average methylation percentage of the first three CpG sites was considered and compared between sexes (F vs M) and conditions (AD vs HC) (Supplementary Fig. 2). Both for PBMCs and for HIC brain tissue, no differences were observed between sexes in AD nor in HC in both R1 and R2 (Supplementary Fig. 2A, B, C, D) (Supplementary Table 7).

Lastly, also the expression of *APOE* was considered to see whether it changed between the two conditions. Given the little to no expression of *APOE* obtained in PBMCs, also supported by The Human Protein Atlas database [32], only brain tissue samples were considered and a not significant increase in *APOE* mRNA levels was observed in AD patients with respect to HC (Supplementary Fig. 3) (Supplementary Table 8).

### Correlation between 2^CpG2^ methylation and CSF biomarkers

**Figure 6.**
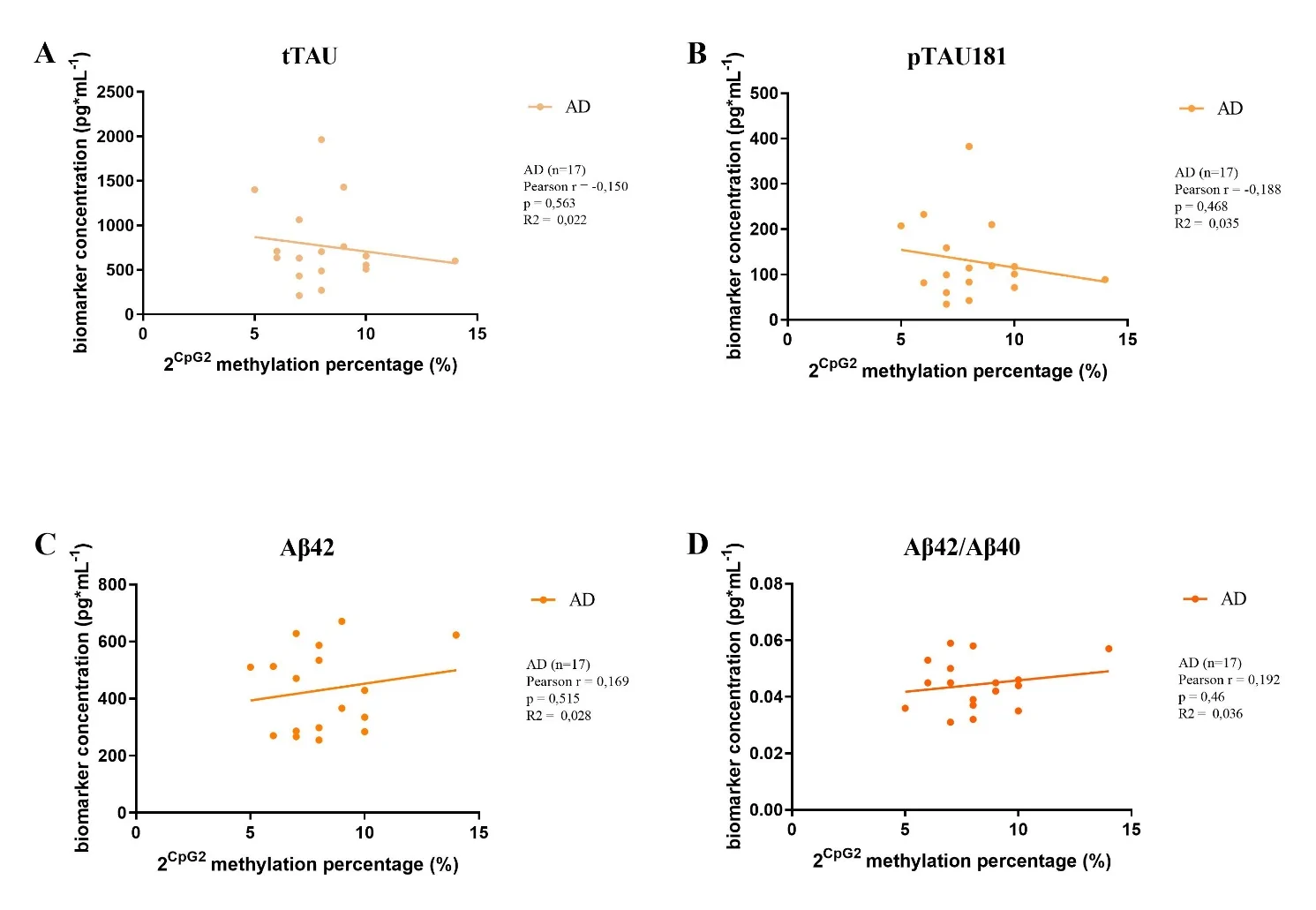
**Correlation between 2^CpG2^ methylation and CSF tTAU, pTAU181, Aβ42 and Aβ42/Aβ40 levels**. (**A**) tTAU CSF concentration on 2^CpG2^ methylation level in AD group (n=17). (**B**) pTAU181 CSF concentration on 2^CpG2^ methylation level in AD group (n=17). (**C**) Aβ42 CSF concentration on 2^CpG2^ methylation level in AD group (n=17). (**D**) Aβ42/Aβ40 CSF concentration on 2^CpG2^ methylation level in AD group (n=17). X axis: 2^CpG2^ methylation percentage; Y axis: biomarker concentration. The relationship between 2^CpG2^ site methylation and CSF biomarkers level was assessed by Pearson’s correlation coefficient and R square (R^2^).

Since 2^CpG2^ site methylation has been described to be correlated with CSF level of tTAU and pTAU181 in patients presenting a Mild-Cognitive Impairment (MCI) [28], the correlation between this site methylation and the concentration of CSF tTAU, pTAU181, Aβ42 and Aβ42/Aβ40 ratio was explored (Fig. 6). CSF biomarkers measurements were available for only 17 AD patients recruited at Mondino Foundation. The results showed that in AD patients the levels of tTAU and pTAU181 appeared to be inversely correlated to 2^CpG2^ methylation (tTAU: Pearson r=-0.150; p=0.563; R^2^=0.022; pTAU181: Pearson r=-0.188; p=0.468; R^2^=0.035) (Fig. 6A, B). At the same time, 2^CpG2^ methylation was found to increase with increasing Aβ42 and Aβ42/Aβ40 ratio, both parameters supporting a control condition (Aβ42: Pearson r=0.169; p=0.515; R^2^=0.028; Aβ42/Aβ40: Pearson r=0.192; p=0.46; R^2^=0.036) (Fig. 6C, D).

## DISCUSSION

The majority of AD cases occur at a late stage of life, with symptoms onset at age equal or over 65 years [1], and these forms of LOAD are associated with several risk factors [4], the most important of which is aging [5] followed by the genetic factors [6]. Among this last category, *APOE* gene is of great interest since it has been reported to be the most influential genetic risk factor [10,33]. Indeed, the combination of two SNPs present on *APOE* exon 4 gives rise to three different protein isoforms [14], one of which (ApoE4) was described to negatively affect Aβ clearance [34,35] due to a higher binding affinity with Aβ [36], hence contribute to the progressive spread of amyloid plaques through neocortex, allocortex, basal ganglia and then brainstem and cerebellum [37]. During the last decade, a significant number of studies have reported an association between AD pathology and aberrant methylation [38]. Different genes have been described to be differentially methylated in AD patients brain tissue, among them *PM20D1* [39], *TREM2* [21], *ANK1* [22], *APP* and *MAPT* [23]. Other regions were observed to present an altered methylation also in peripheral blood, for instance the D-loop region in mitochondrial DNA [40], *COASY* promoter region [41] and also *APP*, *APOE* and *TOMM40* genes [25]. Considering the major role played in the disease, *APOE* methylation has been investigated in the past years [17,26]. Interestingly, *APOE* CpG island methylation was found to be lower than healthy control subjects in a genotype-dependent way in areas of the brain strongly impacted by the disease [17]. As for the promoter region, different studies observed a hypomethylation in patients’ brain tissues that has also been confirmed in lymphocytes [27,28].

To our knowledge, this is the first study that aims to identify a putative differential methylation in *APOE* 5’UTR using the pyrosequencing technique and comparing data obtained at the peripheral level (blood) with data coming from the central nervous system (hippocampus brain tissue) of AD patients. The cohort used in this analysis is composed by Northern Italian AD patients showing a typical pathological phenotype and a similar symptomatic spectrum, as supported by the homogeneity of the obtained results. *APOE* 5’UTR region was divided into two smaller fractions, R1 (chr19:44,905,989-44,906,176, GRCh38/hg38) and R2 (chr19:44,906,058-44,906,285, GRCh38/hg38) for technical limitations, and their methylation pattern was analyzed in PBMCs of 40 subjects (AD n = 20; HC n = 20) and in HIC brain tissue of 9 subjects (AD n = 6; HC n = 3). Coherently with literature, this gene-specific analysis underlined a generalized hypomethylation in both R1 and R2 of *APOE* 5’UTR, in both PBMCs and HIC tissues of AD patients [28,42,43]. In R1, 1^CpG3^ showed an increased methylation level in AD in both PBMCs and HIC. Furthermore, R2 2^CpG2^ displayed a higher methylation level in HC samples of both tissue types. Despite the absence of a statistical significance in this exploratory study, 2^CpG2^ has been investigated in another study and its methylation was also observed to be higher in HIC and cerebellum of healthy control subjects with respect to AD patients [28]. The same study also correlated 2^CpG2^ methylation level with tTAU and pTAU181 levels in CSF of MCI patients, a symptomatic stage that usually precedes AD onset [28]. This correlation was tested also in the AD cohort used in this study and the 2^CpG2^ site methylation further highlighted a certain level of association with CSF biomarkers concentration, with an increase of its methylation level being related to a decrease in tTAU and pTAU181 and to an increase in Aβ42 and Aβ42/Aβ40 ratio, conditions closest to a normal clinical picture. The limited number of samples employed in this study, especially for brain tissue, prevents from appreciating a strong statistical analysis that could support what observed. Furthermore, the homogeneous geographical origin of the selected cohort as well as the patients’ strict selection criteria also preclude the possibility to generalize the obtained data to broader populations or to wider AD or other neurodegenerative disorders’ cohorts. Lastly, it is worth mentioning that different parameters such as education, family history, lifestyle, eating behavior and therapy all might affect the methylation pattern creating inter-individual variability and influencing the final analysis readout.

The investigation of ε4 allele effect on the methylation level of the two regions in PBMCs seemed to hint at a possible role played by ε4 on *APOE* 5’UTR methylation given that both in AD and in HC the presence of the allele determined a mild increase or decrease in the methylation within the same condition. Furthermore, *APOE* ε4^-/+^ genotype effect on R1 and R2 methylation in PBMCs revealed a general increase in methylation in AD, with the only exception of 2^CpG2^ that confirmed the previously described trend also in this case. Nevertheless, in order to assess whether ε4 effectively plays a role on *APOE* 5’UTR methylation it is mandatory to perform this analysis on a wider and demographically different cohort. A study has reported the association between the increase in methylation of two *APOE* CpG sites with ε4 allele carriage [44]. These two CpG sites are located in *APOE* intron 2 and CpG island, hence outside the region investigated in this analysis [44].

Age was also taken into consideration and studied in relation to *APOE* 5’UTR methylation. Notably, in both tissues, R1 methylation appeared to increase with age in AD and decrease in HC, hence highlighting an opposite trend between the pathological and the normal aging condition. HC group mean age is lower than the AD group one, due to HC cohort being composed by volunteers majorly selected for parameters such as healthy neurological condition and absence of pharmacological therapy, both being factors that might determine a change in methylation patterns.

The association between age and methylation alterations has been previously described in a methylome study that identified multiple alterations linked not only to age but also to dementia status and that resulted altered already in pre-diagnostic stages of the disease [45].

As previously mentioned, given the complexity of AD pathology, providing the correct diagnosis is often challenging. For this reason, different cognitive tests are used by physicians as valuable tools to help deliver the correct clinical picture. Among these tests, the MMSE is the most widely applied one [46]. By analyzing MMSE relation with *APOE* 5’UTR methylation, for both regions, it was observed that the average methylation decreased with increasing MMSE cognitive score, thus it appeared inversely related to the level of cognitive function. Finally, *APOE* 5’UTR methylation percentage was also compared between sexes although no differences were observed in both tissues.

In conclusion, in this study, for the first time *APOE* 5’UTR methylation has been analyzed in both peripheral blood and brain tissue using a high in-depth technique, allowing the investigation of every CpG site methylation level. This region resulted to be hypomethylated in both AD and HC conditions. 2^CpG2^ methylation was proved to be particularly decreased in AD confirming data from literature, and it was the only site to maintain this trend even when patient’s *APOE* genotype was taken into consideration. Moreover, ε4 allele highlighted a possible link to a stronger methylation effect in AD in the analyzed region, although in a not significant way. 2^CpG2^ site methylation profile further suggested a putative association with CSF biomarkers level. Indeed, patients with higher 2^CpG2^ methylation score, resembling HC ones, also presented CSF biomarkers levels closest to the normal clinical condition. Finally, increasing age appeared to be related to a higher methylation level in the analyzed region in AD pathological condition, while the opposite trend was observed in HC. However, further studies are required to better describe the methylation distribution in AD pathology, also considering wider cohorts and other *APOE* regions to identify the link between changes in specific sites methylation and other AD specific biomarkers.

### Limitations

The lack of a significant statistical analysis supporting these data can be addressed by considering not only the limited sample size used for this study, especially for brain tissues, but also the small investigated region itself, as well as the selected tissues [47]. Furthermore, the homogeneous demographic origin and the parameters employed to select the cohort used in this study prevents from any attempt to generalize the observed data to other populations. Indeed, *APOE* methylation has been described as tissue and cell specific [17,48], hence the starting material could influence the observed methylation pattern.

## Supplementary Data

The Supplementary data can be found online at: www.aginganddisease.org/EN/10.14336/AD.2024.0350.
