# Supplementary material for: APOE 5’UTR Methylation Pattern Analysis in Blood and Brain Tissue from Alzheimer’s Disease Affected Patients
Source: Aging Dis. 2024 Jun 24;16(3):1639–51. doi: 10.14336/AD.2024.0350 (PMC12096928; doi:10.14336/AD.2024.0350)
Supplement: Supplementary file 1 — The Supplementary data can be found online at: www.aginganddisease.org/EN/10.14336/AD.2024.0350. [file AD-16-3-1639-s.pdf]

SUPPLEMENTARY DATA

APOE 5’UTR Methylation Pattern Analysis in Blood and Brain Tissue from Alzheimer’s Disease Affected Patients

Rosalinda Di Gerlando, Francesca Dragoni, Bartolo Rizzo, Riccardo Rocco Ferrari, Elisabetta Zardini, Matteo Cotta Ramusino, Giulia Perini, Alfredo Costa, Tino Emanuele Poloni, Orietta Pansarasa, Annalisa Davin, Stella Gagliardi

© 2024. Di Gerlando R et al. Published online at http://www.aginganddisease.org/EN/10.14336/AD.2024.0350

SUPPLEMENTARY DATA

SUPPLEMENTARY MATERIALS:

Supplementary figures

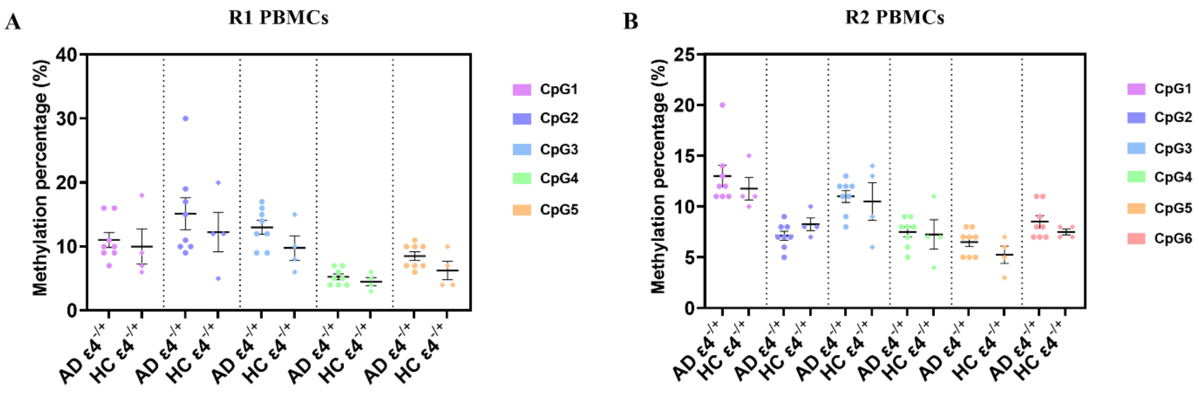

Supplementary Figure 1. *APOE* ε4$^{-/+}$ genotype effect on methylation level of single CpG sites in *APOE* 5'UTR in PBMCs. (**A**) R1 methylation level in AD ε4$^{-/+}$ (n=8) and HC ε4$^{-/+}$ (n=4) PBMCs. (**B**) R2 methylation level in AD ε4$^{-/+}$ (n=8) and HC ε4$^{-/+}$ (n=4) PBMCs. X axis: condition and *APOE* genotype. Single CpG sites are differentiated by color. AD and HC groups are differentiated by symbol (● = AD; ♦ = HC). Statistical analysis was performed using the one-way ANOVA Kruskal-Wallis test comparing AD ε4$^{-/+}$ with HC ε4$^{-/+}$ methylation percentage site by site and data are expressed as mean ± SEM. Data are not significant (Supplementary Table 6).

© 2024. Di Gerlando R et al. Published online at http://www.aginganddisease.org/EN/10.14336/AD.2024.0350

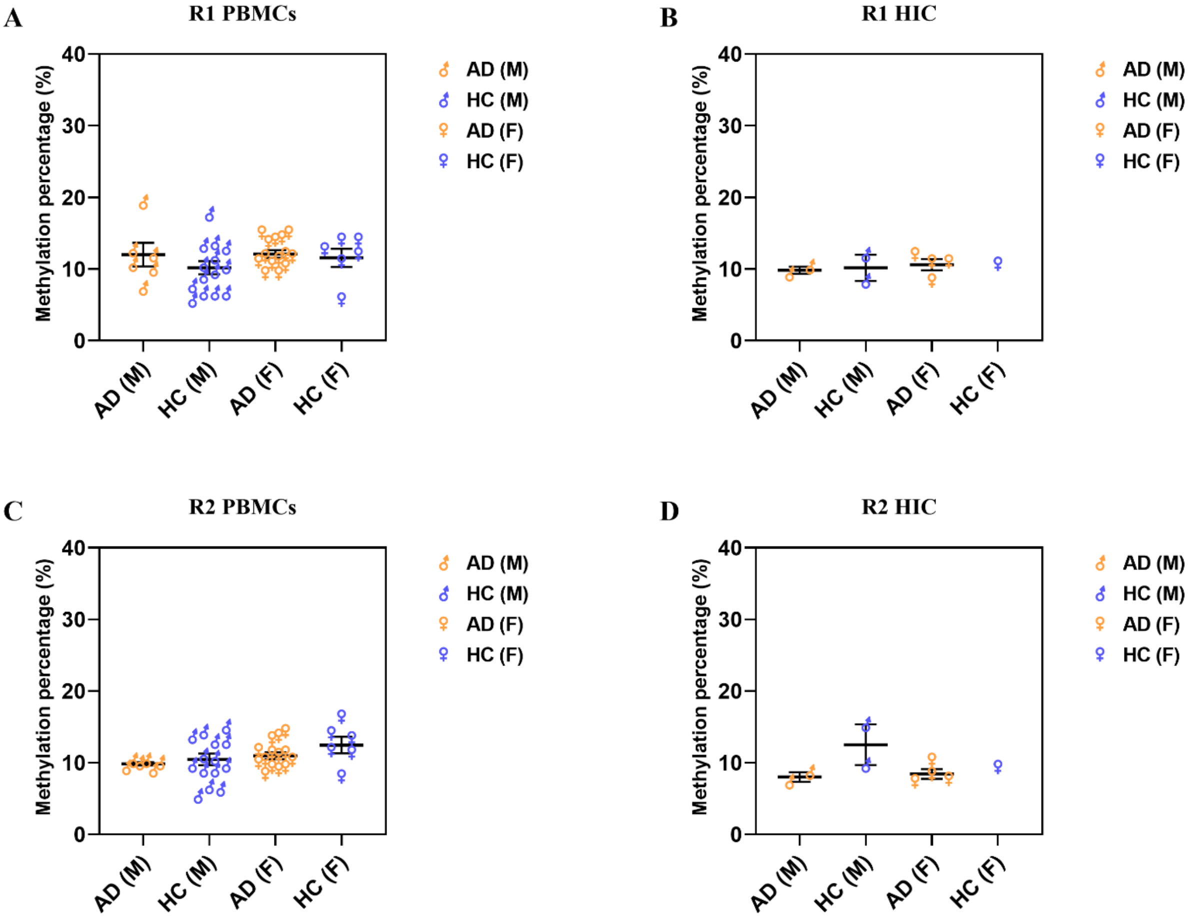

Supplementary Figure 2. Sex effect on the average methylation level of the two regions comprised in *APOE* 5'UTR of both PBMCs and HIC brain tissue. (**A**) R1 average methylation level in AD (M n=6; F n=16) and HC (M n=16; F n=6) PBMCs. (**B**) R1 average methylation level in AD (M n=2; F n=6) and HC (M n=2; F n=1) HIC brain tissue. (**C**) R2 average methylation level in AD (M n=6; F n=16) and HC (M n=16; F n=6) PBMCs. (**D**) R2 average methylation level in AD (M n=2; F n=6) and HC (M n=2; F n=1) HIC brain tissue. X axis: condition and sex; Y axis: average methylation percentage of the first three CpG sites. Females and males are differentiated by symbol (♀ = female; ♂ = male). AD and HC are differentiated by color. Statistical analysis was performed using the one-way ANOVA Kruskal-Wallis test comparing all variables and data are expressed as mean ± SEM. Data are not significant (Supplementary Table 7).

© 2024. Di Gerlando R et al. Published online at http://www.aginganddisease.org/EN/10.14336/AD.2024.0350

SUPPLEMENTARY DATA

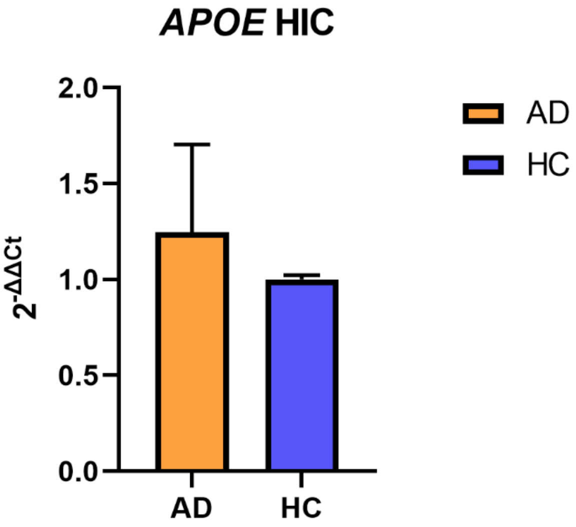

Supplementary Figure 3. *APOE* expression analysis by Real Time PCR in HIC brain tissue of AD patients (n=6) and HC (n=3). X axis: condition; Y axis: Fold-expression indicated as $2^{-\Delta\Delta Ct}$. Statistical analysis was performed using the Student's t test and data are expressed as mean ± SEM. Data are not significant (Supplementary Table 8).

© 2024. Di Gerlando R et al. Published online at http://www.aginganddisease.org/EN/10.14336/AD.2024.0350

SUPPLEMENTARY DATA

Supplementary Tables

Blood sample (IRCCS Mondino Foundation, Pavia)

						CSF			
ID	***Dx***	***Age***	***Sex***	***MMSE***	***APOE genotype***	***tTAU (pg/mL)***	***pTAU181 (pg/mL)***	***Aβ42 (pg/mL)***	***Aβ42/Aβ40***
ADP1	AD	77	F	19	3//4	1064	159	286	0,031
ADP2	AD	80	F	NA	2//3	657	117,5	335	0,035
ADP3	AD	50	F	23,3	3//3	1431	210,2	671	0,045
ADP4	AD	68	F	26,5	3//4	1964	382,6	534	0,032
ADP5	AD	72	M	21	3//4	1402	207,5	510	0,036
ADP6	AD	69	M	20	3//3	709	232,5	270	0,045
ADP7	AD	56	M	26	3//3	NA	NA	NA	NA
ADP8	AD	84	M	19	3//3	NA	NA	NA	NA
ADP9	AD	77	F	14	3//4	271	42,5	255	0,039
ADP10	AD	80	F	12	3//3	509	71,3	284	0,044
ADP11	AD	65	F	10	3//4	636	81,9	513	0,053
ADP12	AD	76	F	28,8	3//4	632	99,2	471	0,045
ADP13	AD	77	M	29,2	3//3	489	83,5	587	0,058
ADP14	AD	77	M	19	3//4	432	60,3	628	0,059
ADP15	AD	68	F	15,9	3//4	761	119,3	366	0,042
ADP16	AD	84	F	16	3//3	NA	NA	NA	NA
ADP17	AD	72	F	17	3//3	600	89	623	0,057
ADP18	AD	81	F	23	3//3	555	101	429	0,046
ADP19	AD	69	F	20	3//3	705	114,3	298	0,037
ADP20	AD	67	F	17	2//3	212	34,8	267	0,050
HCP1	HC	69	M	NA	3//3	NA	NA	NA	NA
HCP2	HC	62	M	NA	3//3	NA	NA	NA	NA
HCP3	HC	41	F	NA	3//3	NA	NA	NA	NA
HCP4	HC	60	M	NA	3//3	NA	NA	NA	NA
HCP5	HC	60	M	NA	3//4	NA	NA	NA	NA
HCP6	HC	51	F	NA	3//4	NA	NA	NA	NA
HCP7	HC	64	M	NA	3//3	NA	NA	NA	NA
HCP8	HC	61	M	NA	3//3	NA	NA	NA	NA
HCP9	HC	59	F	NA	2//3	NA	NA	NA	NA
HCP10	HC	63	M	NA	3//3	NA	NA	NA	NA
HCP11	HC	64	M	NA	3//3	NA	NA	NA	NA
HCP12	HC	67	F	NA	3//3	NA	NA	NA	NA
HCP13	HC	58	M	NA	3//4	NA	NA	NA	NA
HCP14	HC	65	F	NA	3//3	NA	NA	NA	NA
HCP15	HC	65	M	NA	3//4	NA	NA	NA	NA
HCP16	HC	60	M	NA	3//3	NA	NA	NA	NA
HCP17	HC	55	F	NA	3//3	NA	NA	NA	NA
HCP18	HC	57	M	NA	3//3	NA	NA	NA	NA
HCP19	HC	57	M	NA	3//3	NA	NA	NA	NA
HCP20	HC	64	M	NA	3//3	NA	NA	NA	NA

Supplementary Table 1. Clinical parameters of IRCCS Mondino Foundation recruited subjects. ID = sample identification number (ADP = Alzheimer's Disease PBMCs; HCP = Healthy Control PBMCs). Dx = diagnosis (AD = Alzheimer's Disease; HC = Healthy Control). MMSE = Mini-Mental State Examination. F = female. M = male. CSF = cerebrospinal fluid (tTAU = total TAU; pTAU181 = TAU protein phosphorylated at residue 181). NA = not available.

SUPPLEMENTARY DATA

Hippocampus tissue samples (Golgi Cenci Foundation, Abbiategrasso)

ID	*Dx*	*Age*	*Sex*	*MMSE*	*APOE genotype*	*Braak stage*	*Thal phase*
ADH1	AD	80	M	4	3//3	VI	5
ADH2	AD	80	M	0	3//3	V	4
ADH3	AD	89	F	0	3//4	V	5
ADH4	AD	85	F	2	3//3	V	5
ADH5	AD	78	F	0	2//3	IV-V	4
ADH6	AD	82	F	20,7	3//4	III	4
HCH1	HC	81	M	28	2//3	0	0
HCH2	HC	71	F	30	3//3	I+	0
HCH3	HC	79	M	25,3	3//3	I	1

Supplementary Table 2. Clinical parameters of Golgi Cenci Foundation recruited subjects. ID = sample identification number (ADH = Alzheimer's Disease Hippocampus; HCH = Healthy Control Hippocampus). Dx = diagnosis (AD = Alzheimer's Disease; HC = Healthy Control). F = female. M = male. MMSE = Mini-Mental State Examination.

Methylation analysis of single CpG sites in the two regions comprised in APOE 5'UTR of both PBMCs and HIC brain tissue (Figure 1)

Figure	*Comparison*	*Statistical test*	*Significance*	*Adjusted p-value*
1A	1^{CpG1} AD vs HC	One-way ANOVA Kruskal-Wallis test	NS	>0,9999
	1^{CpG2} AD vs HC		NS	>0,9999
	1^{CpG3} AD vs HC		NS	>0,9999
	1^{CpG4} AD vs HC		NS	>0,9999
	1^{CpG5} AD vs HC		NS	>0,9999
1B	1^{CpG1} AD vs HC	One-way ANOVA Kruskal-Wallis test	NS	>0,9999
	1^{CpG2} AD vs HC		NS	>0,9999
	1^{CpG3} AD vs HC		NS	>0,9999
	1^{CpG4} AD vs HC		NS	>0,9999
	1^{CpG5} AD vs HC		NS	>0,9999
1C	2^{CpG1} AD vs HC	One-way ANOVA Kruskal-Wallis test	NS	>0,9999
	2^{CpG2} AD vs HC		NS	>0,9999
	2^{CpG3} AD vs HC		NS	>0,9999
	2^{CpG4} AD vs HC		NS	>0,9999
	2^{CpG5} AD vs HC		NS	>0,9999
	2^{CpG6} AD vs HC		NS	>0,9999
1D	2^{CpG1} AD vs HC	One-way ANOVA Kruskal-Wallis test	NS	>0,9999
	2^{CpG2} AD vs HC		NS	>0,9999
	2^{CpG3} AD vs HC		NS	>0,9999
	2^{CpG4} AD vs HC		NS	>0,9999
	2^{CpG5} AD vs HC		NS	>0,9999
	2^{CpG6} AD vs HC		NS	>0,9999

Supplementary Table 3. Details on the statistical analysis with relative p-values. AD = Alzheimer's disease; HC = Healthy Control; $CpG^{\#}$ = CpG number #; NS = not significant.

Average methylation level of the two regions comprised in APOE 5'UTR of both PBMCs and HIC brain tissue (Figure 2)

Figure	*Comparison*	*Statistical test*	*Significance*	*Adjusted p-value*
2A	AD vs HC	Student's t Mann-Whitney test	NS	0,1889
2B	AD vs HC	Student's t Mann-Whitney test	NS	>0,9999
2C	AD vs HC	Student's t Mann-Whitney test	NS	0,5506
2D	AD vs HC	Student's t Mann-Whitney test	NS	0,0833

© 2024. Di Gerlando R et al. Published online at http://www.aginganddisease.org/EN/10.14336/AD.2024.0350

SUPPLEMENTARY DATA

Supplementary Table 4. Details on the statistical analysis with relative p-values.
AD = Alzheimer's disease; HC = Healthy Control; NS = not significant.

APOE ε4 allele effect on average methylation level of the two regions in PBMCs (Figure 3)

Figure	*Comparison*	*Statistical test*	*Significance*	*Adjusted p-value*
3A	AD $\varepsilon4^{-/-}$ vs AD $\varepsilon4^{-/+}$	One-way ANOVA Kruskal-Wallis test	NS	>0,9999
	AD $\varepsilon4^{-/-}$ vs HC $\varepsilon4^{-/-}$		NS	>0,9999
	AD $\varepsilon4^{-/-}$ vs HC $\varepsilon4^{-/+}$		NS	>0,9999
	AD $\varepsilon4^{-/+}$ vs HC $\varepsilon4^{-/-}$		NS	0,7518
	AD $\varepsilon4^{-/+}$ vs HC $\varepsilon4^{-/+}$		NS	0,7565
	HC $\varepsilon4^{-/-}$ vs HC $\varepsilon4^{-/+}$		NS	>0,9999
3B	AD $\varepsilon4^{-/-}$ vs AD $\varepsilon4^{-/+}$	One-way ANOVA Kruskal-Wallis test	NS	>0,9999
	AD $\varepsilon4^{-/-}$ vs HC $\varepsilon4^{-/-}$		NS	>0,9999
	AD $\varepsilon4^{-/-}$ vs HC $\varepsilon4^{-/+}$		NS	>0,9999
	AD $\varepsilon4^{-/+}$ vs HC $\varepsilon4^{-/-}$		NS	>0,9999
	AD $\varepsilon4^{-/+}$ vs HC $\varepsilon4^{-/+}$		NS	>0,9999
	HC $\varepsilon4^{-/-}$ vs HC $\varepsilon4^{-/+}$		NS	>0,9999

Supplementary Table 5. Details on the statistical analysis with relative p-values.
AD = Alzheimer's disease; HC = Healthy Control; NS = not significant.

APOE $\varepsilon4^{-/+}$ genotype effect on methylation level of single CpG sites in APOE 5'UTR in PBMCs (Supplementary Figure 1)

Figure	*Comparison*	*Statistical test*	*Significance*	*Adjusted p-value*
S1A	1^{CpG1} AD $\varepsilon4^{-/+}$ vs HC $\varepsilon4^{-/+}$	One-way ANOVA Kruskal-Wallis test	NS	>0,9999
	1^{CpG2} AD $\varepsilon4^{-/+}$ vs HC $\varepsilon4^{-/+}$		NS	>0,9999
	1^{CpG3} AD $\varepsilon4^{-/+}$ vs HC $\varepsilon4^{-/+}$		NS	>0,9999
	1^{CpG4} AD $\varepsilon4^{-/+}$ vs HC $\varepsilon4^{-/+}$		NS	>0,9999
	1^{CpG5} AD $\varepsilon4^{-/+}$ vs HC $\varepsilon4^{-/+}$		NS	>0,9999
S1B	2^{CpG1} AD $\varepsilon4^{-/+}$ vs HC $\varepsilon4^{-/+}$	One-way ANOVA Kruskal-Wallis test	NS	>0,9999
	2^{CpG2} AD $\varepsilon4^{-/+}$ vs HC $\varepsilon4^{-/+}$		NS	>0,9999
	2^{CpG3} AD $\varepsilon4^{-/+}$ vs HC $\varepsilon4^{-/+}$		NS	>0,9999
	2^{CpG4} AD $\varepsilon4^{-/+}$ vs HC $\varepsilon4^{-/+}$		NS	>0,9999
	2^{CpG5} AD $\varepsilon4^{-/+}$ vs HC $\varepsilon4^{-/+}$		NS	>0,9999
	2^{CpG6} AD $\varepsilon4^{-/+}$ vs HC $\varepsilon4^{-/+}$		NS	>0,9999

Supplementary Table 6. Details on the statistical analysis with relative p-values.
AD = Alzheimer's disease; HC = Healthy Control; $CpG^{\#}$ = CpG number #; NS = not significant.

Sex effect on the average methylation level of the two regions comprised in APOE 5'UTR of both PBMCs and HIC brain tissue (Supplementary Figure 2)

Figure	*Comparison*	*Statistical test*	*Significance*	*Adjusted p-value*
S2A	AD (M) vs HC (M)	One-way ANOVA Kruskal-Wallis test	NS	>0,9999
	AD (M) vs AD (F)		NS	>0,9999
	AD (M) vs HC (F)		NS	>0,9999
	HC (M) vs AD (F)		NS	0,4191
	HC (M) vs HC (F)		NS	>0,9999
	AD (F) vs HC (F)		NS	>0,9999
S2B	AD (M) vs HC (M)	One-way ANOVA Kruskal-Wallis test	NS	>0,9999
	AD (M) vs AD (F)		NS	>0,9999
	AD (M) vs HC (F)		NS	>0,9999
	HC (M) vs AD (F)		NS	>0,9999
	HC (M) vs HC (F)		NS	>0,9999
	AD (F) vs HC (F)		NS	>0,9999

© 2024. Di Gerlando R et al. Published online at http://www.aginganddisease.org/EN/10.14336/AD.2024.0350

S2C	AD (M) vs HC (M)	One-way ANOVA Kruskal-Wallis test	NS	>0,9999
	AD (M) vs AD (F)		NS	>0,9999
	AD (M) vs HC (F)		NS	0,3312
	HC (M) vs AD (F)		NS	>0,9999
	HC (M) vs HC (F)		NS	0,7159
	AD (F) vs HC (F)		NS	>0,9999
S2D	AD (M) vs HC (M)	One-way ANOVA Kruskal-Wallis test	NS	0,4893
	AD (M) vs AD (F)		NS	>0,9999
	AD (M) vs HC (F)		NS	>0,9999
	HC (M) vs AD (F)		NS	0,6052
	HC (M) vs HC (F)		NS	>0,9999
	AD (F) vs HC (F)		NS	>0,9999

Supplementary Table 7. Details on the statistical analysis with relative p-values.
AD = Alzheimer's disease; HC = Healthy Control; NS = not significant.

APOE expression analysis by Real Time PCR in HIC brain tissue (Supplementary Figure 3)

Figure	***Comparison***	***Statistical test***	***Significance***	***Adjusted p-value***
S3	AD vs HC	Student’s t Mann-Whitney test	NS	0,7143

Supplementary Table 8. Details on the statistical analysis with relative p-values.
AD = Alzheimer's disease; HC = Healthy Control; NS = not significant.

© 2024. Di Gerlando R et al. Published online at http://www.aginganddisease.org/EN/10.14336/AD.2024.0350
